# Horizontal Honey-Bee Larvae Rearing Plates Can Increase the Deformation Rate of Newly Emerged Adult Honey Bees

**DOI:** 10.3390/insects12070603

**Published:** 2021-07-01

**Authors:** Juyeong Kim, Kyongmi Chon, Bo-Seon Kim, Jin-A Oh, Chang-Young Yoon, Hong-Hyun Park, Yong-Soo Choi

**Affiliations:** 1 Toxicity and Risk Assessment Division, Department of Agro-food Safety and Crop Protection, National Institute of Agricultural Sciences, Rural Development Administration, Wanju-gun 55365, Korea; kjy.sara@gmail.com (J.K.); kbs9249@naver.com (B.-S.K.); oja5074@korea.kr (J.-A.O.); evermoo2600@korea.kr (C.-Y.Y.); honghyunpark@korea.kr (H.-H.P.); 2 Sericulture and Apiculture Division, Department of Agricultural Biology, National Institute of Agricultural Sciences, Rural Development Administration, Wanju-gun 55365, Korea; beechoi@korea.kr

**Keywords:** *Apis mellifera*, deformation, emergence, honey bee, in vitro rearing, larvae

## Abstract

**Simple Summary:**

Rearing honey bee (*Apis mellifera*) larvae in vitro is an important method for studying bee larvae diseases or the toxicity of pesticides on bees. Laboratory experiments for bee larvae are usually performed by placing a rearing plate horizontally during all developmental stages. However, recent studies have demonstrated that a horizontal rearing environment can cause the deformation of emerged bees. Most studies adopted a vertical rearing method to reduce such deformation, but there is a lack of information on the emergence rates and deformation rates of bees reared on vertical or horizontal plates. Therefore, in this study, we examined the effect of placing the plates vertically and horizontally on newly emerged bees. There were no significant differences in larval mortality, pupal mortality, and adult emergence rates between horizontal and vertical rearing plates. However, the adult deformation rates of the horizontal plates were significantly higher than those of the vertical plates. In conclusion, we suggest that the vertical rearing method is more suitable when considering the deformation rate of the control group to verify the sublethal effects of pesticides on honey bees.

**Abstract:**

Rearing honey bee larvae in vitro is an ideal method to study honey bee larval diseases or the toxicity of pesticides on honey bee larvae under standardized conditions. However, recent studies reported that a horizontal position may cause the deformation of emerged bees. Accordingly, the purpose of this study was to evaluate the emergence and deformation rates of honey bee (*Apis mellifera ligustica*) larvae reared in horizontal and vertical positions. The study was conducted under the same laboratory conditions with three experimental groups, non-capped or capped horizontal plates and capped vertical plates. However, our results demonstrated that the exhibited adult deformation rates of the horizontal plates were significantly higher (27.8% and 26.1%) than those of the vertical plates (11.9%). In particular, the most common symptoms were deformed wings and an abnormal abdomen in the horizontal plates. Additionally, adults reared on horizontal plates were substantially smaller (10.88 and 10.82 mm) than those on vertical plates (11.55 mm). Considering these conclusions, we suggest that a vertical rearing method is more suitable when considering the deformation rates of the control groups to verify the sublethal effects of pesticides on honey bees.

## 1. Introduction

Many studies have reported recent significant pollinator declines and increased honey bee (*Apis mellifera*) colony losses in many countries [1,2,3,4,5]. Several stressing factors, such as pathogens, climate change, parasites, habitat loss, lack of nutrition, pesticides, and diseases are considered to explain the decline and colony losses [6,7,8,9]. However, a single causative stressor factor has not been conclusively identified, because of the complexity related to concurrent multiple stressors [4,10,11,12]. Among the several factors suggested, pesticides are regarded as one of the most crucial causes of adverse honey-bee health and colony declines [4,13,14,15]. Various studies have been conducted to determine the exposure effects of pesticides on adult honey bees, therefore, standard methods for investigating the effects of pesticides on adult bees have been well investigated both in vivo and in vitro [15,16]. Nevertheless, in contrast to controllable laboratory conditions, field experiments in hives are impacted by numerous uncontrollable factors such as season, colony genetic variation, climate, and resource availability [17,18,19]. Because of these uncontrolled variables, the in vitro procedure of rearing honey-bee larvae has been proposed to evaluate the toxicity of pesticides on honey-bee broods (larvae, pupae, and adults) [11]. Rearing larvae in vitro is a practical protocol to study larval pathogens, development, and caste differentiation in honey bees [20,21,22,23]. Nevertheless, available data in the publications concerning the lethal and sublethal effects on honey-bee larvae are rather poor compared to adult bees [11,24,25].

In 1933, the first informative report investigating the caste differentiation of queen and worker bees, as well as hand-feeding bee larvae with a diet containing royal jelly in vitro, was published [26]. The larval diet composition was improved and optimized by Rembold and Lackner [23], and Vandenberg and Shimanuki [27]. Vandenberg and Shimanuki [27] further developed methods of rearing one larva per cup and feeding them the correct quantity of diet daily. Wittmann and Engels [28] reported an in vitro rearing method as a risk assessment tool to study the toxicity of pesticides. Davis et al. [29] provided diets containing carbofuran and dimethoate to larvae reared in the laboratory, and Peng et al. [21] utilized rearing honey-bee larvae in vitro for assessing the toxicity of pesticides on honey bees. Most notably, seven laboratories across five different countries performed ring tests according to the improved in vitro methods to assess LD_₅₀_ for acute toxicity of dimethoate in 2005 and 2008 [17]. The ring test participants achieved adult emergence rates greater than 80% in 43% of their control trials and greater than 90% in 17% of their control trials [12,17]. The Organization for Economic Co-operation and Development (OECD) guidelines for the honey-bee larvae toxicity tests under laboratory conditions were published in 2013 (single exposure) and 2016 (repeated exposure), based on the methods that were developed in ring tests [30,31].

Larval mortality and pupal mortality in the natural hive occurred at approximately 15% [32,33]. Therefore, the OECD guidelines specify that the total mortality during larval and pupal developmental stages should not exceed 15% in the controls, otherwise the study is considered invalid [30,31]. Namely, the mortalities of the control groups should be considered for validation of the test [32].

Accordingly, researchers have focused on increasing the survival rate of the larval stage, and there are several examples of these methods. They mainly improved the survival rate of larvae according to the composition of larval food, quality of royal jelly, larval age at grafting, rearing conditions (temperature and relative humidity) in the laboratory, or reducing contact between larvae and fecal materials using absorbents such as Kimwipes (filter paper) [12,32,34,35,36].

However, even if in vitro rearing protocols have been improved over the years, variable (inconsistent) survival rates in the controls of each laboratory have been reported continuously [12,17]. Zhu et al. [37] reported the larval mortalities of controls were approximately 17.5% at D6 after grafting. Additionally, low emergence rates of controls (≤50%) were noted in several experimental studies based upon Aupinel et al. [11,12]. Namely, this means that several research institutes have already performed a larval toxicity test, with control mortalities higher than the OECD guidelines [17,18,22,38]. The inconsistent results across different laboratories may reflect subtle differences in the brood sources and the laboratory conditions, or be related to the effects of the mechanical stress of grafting [12,19,39]. It is also a more common practice to set up the rearing using 48-well tissue plates, with the grafting cell cups placed horizontally, which is according to the OECD guideline (2016). However, some studies have mentioned that the horizontal rearing positions during the developmental stages may lead to the deformation of wings and abdomen (humpback) in emerged bees, because the larvae or pupae can then withstand abnormal vertical positions [32,40,41]. Riessberger-Gallé et al. [40] proposed a method to prevent these deformations, in which a 48-well tissue plate was sealed with a thin wax layer and set vertically as in natural beehives. However, presently, specific information on the emergence rates and deformation rates of the newly emerged honey bees when the rearing plates are placed vertically or horizontally has not yet been reported. Therefore, in this study, we aimed to evaluate the specific difference in the emergence rate and deformation rate of emerged bees when the rearing plates were positioned horizontally or vertically during the pupal developmental stage.

## 2. Materials and Methods

### 2.1. Rearing Honey Bee Larvae In Vitro

The honey-bee larvae (*Apis mellifera ligustica*) were randomly obtained from three healthy colonies at an apiary located at the National Institute of Agricultural Sciences in Korea (35°49′47.0″ N, 127°02′26.0″ E). The source colonies and bees were not treated against *Varroa destructor* for four weeks prior to the experiment. The honey-bee queens were caged on wax combs using queen excluders to lay eggs. The freshly laid eggs were confirmed the next day (24 h after the queens were caged), and after that, the combs containing the hatched first instar larvae (72 h after the queens laid the eggs) were delivered to the laboratory for grafting. Before grafting, the grafting cell cups (Nicotplast, Maisod, France) were disinfected with 70% ethanol and were then used, after UV sterilization for 30 min in a laminar-flow hood. The larval rearing procedure followed OECD No. 239 [31]. On day 1 (D1), 20 μL of larval diet A was loaded into the grafting cell cup, and healthy first instar larvae were transferred into the cell cups of 48-well tissue plates (SPL, Pocheon-si, Korea). During grafting, a clay pack (Caremate, Hwaseong-si, Korea) was preheated in the microwave and the pack was laid underneath 48-well tissue plates to minimize the temperature effect on the larvae [32]. After grafting, the 48-well tissue plates were placed horizontally in a sealed desiccator (Nalgene, Rochester, NY, USA) in a constant temperature incubator (DAIHAN Scientific Co., Wonju-si, Korea) maintained at 35 °C and 95 ± 5% relative humidity (RH) using a saturated solution of potassium sulfate (Junsei, Tokyo, Japan) during the larval stages (D1–D8). The pupal stages (D8–D15) were maintained at 80 ± 5% RH using a saturated solution of sodium chloride (Sigma–Aldrich, St. Louis, MO, USA). In the emergence stages (D15–D21), the 48-well tissue plates were transferred individually into emergence boxes with a 50% sucrose solution, and placed in the incubator to maintain 50% RH and 35 °C.

For larval feeding, d-glucose (Difco, Sparks, NV, USA) and d-fructose (Junsei, Japan) were added to water filtered with a 0.20 μm filter (Sartorius, Göttingen, Germany), and then yeast extract (Bacto, Sparks, NV, USA) was added and mixed. Lastly, the solution was mixed with royal jelly (Haechangol Honey Farm, Yeongwol, Korea) [12]. Following the OECD guidelines, the larval diets included a total of 160 μL of each standardized volume during the six days (excluding D2) of the larval stages, where the larval diet volume and components have been summarized in Table 1. Before feeding the larvae, diets were preheated in an incubator maintained at 35 °C.

### 2.2. Experimental Design

The non-capped and horizontally oriented groups (NHG) were placed in 48-well tissue plates without a wax layer and set horizontally, and the capped and horizontally oriented groups (CHG) were placed in 48-well tissue plates that were capped with the artificial wax layers and set horizontally. Meanwhile, the capped and vertically oriented groups (CVG) were placed in 48-well tissue plates that were capped with the artificial wax layers and set vertically. All groups were not treated with any chemical reagents. The experiments of each group were tested with 4 replicates (36 larvae per plate). Artificial wax layers were prepared by dissolving 4.0 g of pure beeswax. The size of the wax layers was 14 cm × 10 cm × 0.4 mm. In each grafting cell cup of the plate, small orifices were made to allow air exchange. Rearing plates were sealed with the perforated wax layers on D15 after grafting, particularly vertical plates (CVG) placed carefully upright so pupae were facing towards the opening.

### 2.3. Mortality and Abnormal Symptoms

Mortality and abnormal symptoms at each developmental stage were visually observed and recorded every day. After setting the survival rates of the larvae at 100% on D3, larval mortality, and abnormal symptoms were monitored as early death and melanizing death from D4 to D8 (larval stages) [12,30,31]. The larvae were considered as dead when the larval color became dark or they had no motion, and were removed daily from the test plates. On D7, no additional diet was fed, and on D8, the number of larvae with uneaten diets was recorded. From D8 to D21 (pupal stages), pupal mortality was assessed based on failed molt and failed adult molt [12,31,42]. From D16 to D21 (emergence stages), the number of newly emerged bees was observed and recorded daily. Individuals that died after emergence or fully developed bees that stayed in the cell without breaking the wax layer were considered to be emerged bees. All deformation symptoms and morphological characteristics (weight and length) of dead adult bees after emerging were observed and measured immediately. After all deformation symptoms of living emerged bees were observed on D21, the weight and length of the emerged bees were measured. The whole-body length of an adult bee was measured from the tip of its head to the tip of its abdomen. In particular, when a humpback was present in the bee, the total length of the body was measured as it was in the unstretched state. The symptoms of newly emerged adult bees were classified as surviving normal (SN), deformed wings (DW), deformed antennae (DA), and abnormal abdomen shape (AAS) (Table 2) [43]. The larval mortality, pupal mortality, adult emergence rate, and deformation rate were calculated for each group using the following formulae [31]:

Larval mortality = (the number of dead larvae from D3 to D8/the number of larvae on D3) × 100

Pupal mortality = (the number of dead pupae from D8 to D21/the number of pupae on D8) × 100

Adult emergence rate = (the number of emerged bees/the number of larvae on D3) × 100

Deformation rate = (the number of deformed bees/the number of emerged bees) × 100

### 2.4. Statistical Analysis

A statistical analysis of the data was carried out using the SPSS statistical software program (SPSS 20.0 Inc., Chicago, IL, USA). A Pearson’s chi-square test was used to compare larval mortality, pupal mortality, and adult emergence rates among the three groups. A Fisher’s exact test was used to assess differences between the total deformation rates of emerged bees in the three groups. The Kaplan–Meier log-rank test was used to compare the survival curves of each group. The mortality, emergence rates, and deformation rates were expressed as means ± SE. Means ± SE (standard error) were calculated for the four replicate values of each group. The emergence date, adult weight, and length were expressed as means ± SD (standard deviation). The dates of emergence, and the adult weight and length at D21, were tested by the one-way ANOVA and were determined using Tukey’s HSD test to compare the values among the three groups. A *p*-value of <0.05 was considered as a statistically significant difference.

## 3. Results

### 3.1. In Vitro Mortality, Adult Emergence Rates, and Survival

**Figure 1:**
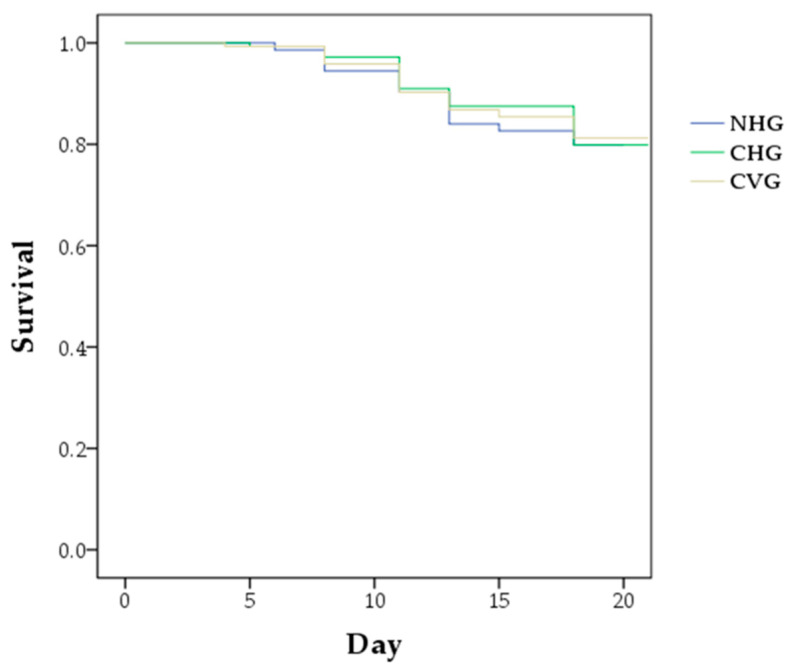
The survival curves of the honey-bee larvae of the horizontally oriented groups and the vertically oriented groups over 21 days, which were assessed by the Kaplan–Meier log-rank test (*p* > 0.05). NHG: the non-capped and horizontally oriented groups; CHG: the capped and horizontally oriented groups; CVG: the capped and vertically oriented groups.

Larval mortality and pupal mortality means ± SE were as follows: 4.9 ± 0.7% and 16.1 ± 2.8%, respectively, in the NHG; 7.7 ± 4.1% and 13.5 ± 3.4%, respectively, in the CHG; and 4.2 ± 2.7% and 15.3 ± 2.2%, respectively, in the CVG. The three groups satisfied the OECD test condition that the larval mortalities were less than 15% in the negative controls. On D21, total emergence rates were 79.9 ± 3.3% in the NHG, 79.9 ± 6.7% in the CHG, and 81.3 ± 0.9% in the CVG, which corresponds to the OECD test condition that the adult emergence rate should be ≥70% in the controls. No statistically significant differences were detected among the three groups with respect to larval mortality, pupal mortality, and adult emergence rates (chi-square test, *p* > 0.05, Table 3). The survival curves of the honey-bee larvae have been illustrated in Figure 1, where no significant differences were found among the three groups in terms of their survival (Kaplan–Meier log-rank test, *p* > 0.05).

### 3.2. Emergence Rates by Time (Days)

**Figure 2:**
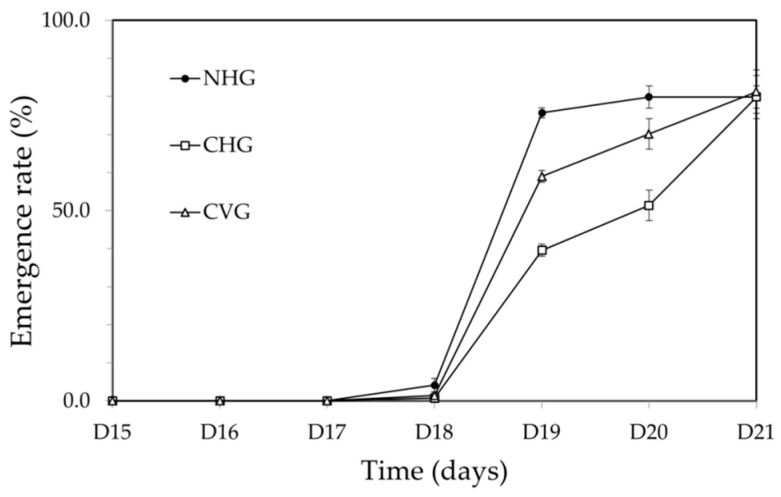
Emergence rates of each group from D15 to D21. NHG: the non-capped and horizontally oriented groups; CHG: the capped and horizontally oriented groups; CVG: the capped and vertically oriented groups.

The emergence rates of each group were assessed according to the time (days). By D17, honey bees from the groups had not emerged, and then, on D18, worker bees began to emerge at 4.0% in the NHG, 0.7% in the CHG, and 1.4% in the CVG. On D19, more than half of the bees (75.7%) emerged in the NHG, and 59.0% in the CVG, compared to the 39.6% of bees that emerged in the CHG. Finally, on D21, 79.9% of bees emerged in the NHG, 79.9% of bees emerged in the CHG, and 81.3% of bees emerged in the CVG (Figure 2). The mean emergence date of the NHG was 19.00 ± 0.32 days, that of the CHG was 19.85 ± 0.93 days, and that of the CVG was 19.39 ± 0.74 days. The three groups demonstrated a statistically significant difference in emergence date (Tukey’s HSD test, *p* < 0.05).

### 3.3. Deformation Rate of Newly Emerged Adult Bees

**Figure 3:**
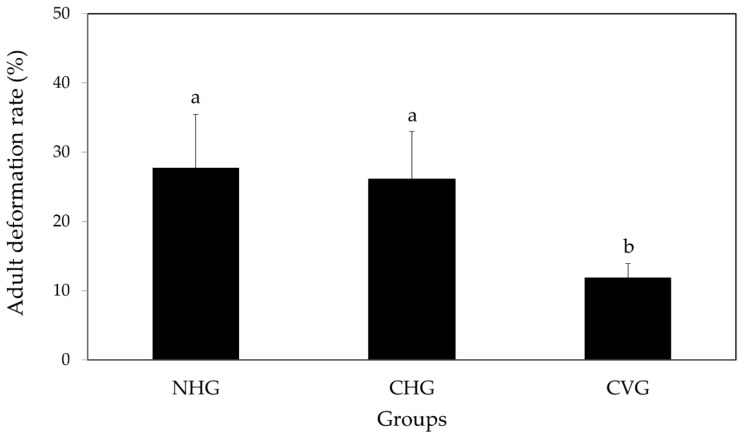
Deformation rates of newly emerged adult bees. The different letters above the bars indicate significant differences among the three groups (Fisher’s exact test, *p* < 0.05). NHG: the non-capped and horizontally oriented groups; CHG: the capped and horizontally oriented groups; CVG: the capped and vertically oriented groups.

**Figure 4:**
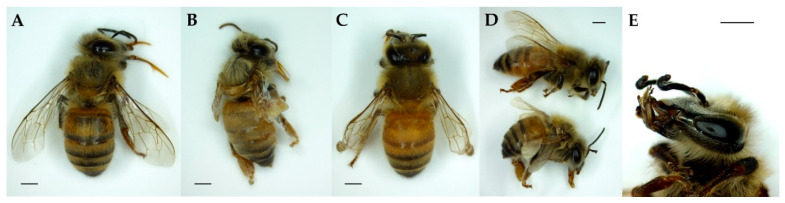
The observed symptoms of newly emerged adult bees. (**A**) Surviving normal (SN); (**B**) Deformed with short wings (DSW); (**C**) Deformed with tangled wings (DTW); (**D**) Normal (above) and abnormal abdomen shape (AAS) (below); (**E**) Deformed antennae (DA). Scale bar = 1 mm.

The total deformation rates were 27.8 ± 7.7% in the NHG, 26.1 ± 6.9% in the CHG, and 11.9 ± 2.0% in the CVG. The deformation rates of NHG and CHG were significantly higher than those of CVG (Fisher’s exact test, *p* < 0.05, Figure 3). Figure 4 illustrates examples of normal and deformed bees in the three groups. DW was shorter or had tangled ends compared to the normal wings. DA had curved ends compared to the normal antennae. Additionally, an AAS was more curved compared to the normal abdomen shape. Deformation symptoms were classified into 9 categories, including AAS, DSW, DTW, deformed with tangled wings asymmetrically (DTWA), and DA. In the NHG, the deformation rates were AAS (13.9%), DSW (0.9%), DTW (0.9%), DTWA (3.5%), AAS + DW (4.3%), DW + DA (0.9%), and AAS + DW + DA (2.6%). In the CHG, the deformation rates were AAS (4.3%), DSW (4.3%), DTW (3.5%), DTWA (6.1%), DA (0.9%), AAS + DW (4.3%), AAS + DA (1.7%), DW + DA (0.9%), and AAS + DW + DA (0.9%). In CVG, the deformation rates were AAS (3.4%), DSW (1.7%), DTW (1.7%), DTWA (3.4%), DA (0.9%), and DW + DA (0.9%) (Table 4).

**Figure 5:**
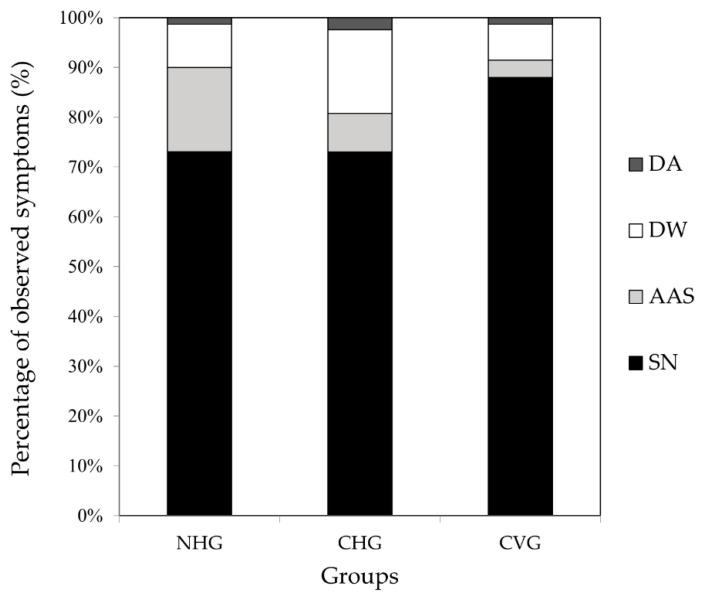
Percentage of observed symptoms of emerged bees. SN: survived and successfully eclosed as bees; AAS: abnormal abdomen shape; DW: deformed wings; DA: deformed antennae.

The main deformation symptoms were simplified into three categories as follows: AAS, DW and DA. The percentage of SN, AAS, DW, and DA were 73.1%, 17.0%, 8.7%, and 1.3%, respectively, in the NHG; 73.0%, 7.7%, 16.8%, and 2.5%, respectively, in the CHG; and 88.0%, 3.4%, 7.3% and 1.3%, respectively, in the CVG (Figure 5).

### 3.4. Body Weight and Length of Newly Emerged Adult Bees

The mean weight and length of the emerged bees were 67.40 ± 12.24 mg and 10.88 ± 0.96 mm in the NHG, 69.77 ± 12.63 mg and 10.82 ± 0.92 mm in the CHG, and 71.95 ± 12.12 mg and 11.55 ± 1.00 mm in the CVG, respectively. Adult weights of CVG were significantly higher than those of NHG (*p* = 0.014). Additionally, the length of the emerged bees in the CVG was significantly larger than that of the NHG and CHG (one-way ANOVA, *F*_(2, 344)_ = 21.072, *p* < 0.05, Table 5).

## 4. Discussion

Many studies have emphasized the importance of rearing honey-bee larvae in vitro for testing the toxicity of pesticides and, sequentially, experimental rearing methods have been systematically developed [12,18,32,44]. Natural or commercial hives consist of several honeybee combs with vertical structures for brood rearing and storing honey-bee products [45]. Conversely, as displayed in the OECD guidelines [30,31], many laboratories have generally performed experiments placing the rearing plates horizontally during all developmental stages (from larvae to adults). However, the horizontal rearing plates may induce deformations in emerged bees [32,41]. The purpose of this study was to analyze the effects of vertical and horizontal rearing plates on the emergence rates and deformation rates of newly emerged bees.

Honey bee larval defecation is usually on D7 after grafting [21]. Several recent studies suggest that the larvae should be transferred to a new clean plate, since larval mortality may increase due to the defecation of the larvae [12,17,38]. However, transferring larvae may cause mechanical stress or contamination of the larvae [39]. Besides, when feeding the total diet of 160 μL, the larvae eat all the food provided, thus, it is not necessary to move the larvae to new cell cups or clean the grafting cell cups [32]. Brodschneider et al. [41] placed rearing plates capped with thin wax layers vertically on D11. As a result, the total mortality until emergence was 16.3% in the control groups; they also reported similar flight performance between reared bees in vitro and hives. Likewise, Krainer et al. [46] demonstrated a total mortality of 28.1% in the control groups. They sealed rearing plates with the wax layer and the plates were placed vertically on D12. In our experiments, there were no significant differences in larval mortality, pupal mortality, and adult emergence rates among the three groups. The total adult emergence rates were about 80% in the three groups. Thus, each group exhibited similar survival rates of bee larvae regardless of plate position (horizontal and vertical).

For the most part, the worker bees reared in vitro emerged on D17–D18 after grafting [20]. In our experiments, bees of each group began to emerge from D18. Bees in the NHG were the earliest to emerge among the three groups. These differences could be due to external stimulus by the newly emerged bees that roam and stimulate other non-emergent pupae in the cells of 48-well tissue plates. Namely, bees in CHG and CVG may emerge more slowly, since the wax layer of the plate could interrupt this external stimulus. In particular, the CHG was the slowest among the three groups. This may be because more force is needed to break through the wax layer against gravity on the horizontal plate than on the vertical plate.

Tehel et al. [47] inoculated the honey-bee pupa with deformed wing virus (DWV) and tested the relative effects of the genotype of DWV on the mortality and wing malformation of adult honey bees. They placed the plate vertically so that pupae were horizontal in the incubators and monitored the pupal development. They observed that 23% of emerged bees in the control had wing deformities. In our experiments, the deformations of adults included deformed wings, deformed antennae, and abnormal abdomen shape, and horizontally oriented groups (NHG and CHG) demonstrated higher deformation rates (27.8% and 26.1%) than the vertically oriented groups (11.9%). In particular, the NHG and CHG had more wing or abdominal deformations than the CVG. Although the cause of deformation in emerged bees is not clear, all adults with deformations were derived only from deformed pupae [43]. Additionally, the wings of honey bees are formed during the pupal development stage [48]. When the rearing plates were horizontal, the pupae in the cell cups hold a vertical state. Thus, the pupal body is pulled down by gravity, affecting the wing and abdominal development [41]. In this regard, deformed bees may already have external deformations from the pupal stage.

In other studies, the rearing plates were capped and set vertically on D11 after grafting when pupation started, but in our experiments, the plates were capped with a wax layer on D15 to observe the mortality during the pupal stages. For this reason, it is thought that the horizontal condition between D11 and D14 (before capping the plates with wax layer) had already affected the pupae, resulting in abdominal and wing deformations in the CVG. Mechanisms for explaining antennae deformations due to physical external deformation have not been described in other studies.

The average adult weight ranged from 67.40 to 71.95 mg in our experiments; similarly, Brodschneider et al. [41] measured 76.6 ± 11.6 mg in emerged bees of the control groups. The average adult weight of the CVG was significantly higher than that of the NGH. The emerged bees of the CVG were significantly larger by 0.7 mm than the NHG and CHG, and this difference appeared to be due to the abnormal shapes, such as the humpback and abdominal shrinkage that were observed in bees reared horizontally.

Barbosa et al. [43] reported that when azadirachtin and spinosad were treated on the stingless bee, *Melipona quadrifasciata*, deformed pupae and emerged bees with wing, antennae, and leg deformities occurred. Additionally, they reported that deformed bees had side effects regarding flight activity or olfactory activity. Therefore, the deformations occurring in emerged bees have the potential to directly affect the activity of worker bees, and thus, these deformations can also be evaluated as sublethal effects [43]. In the present study, the mean adult deformation rates in CVG were approximately 12%. Thus, the vertical rearing method can be supported as a more appropriate method to verify the effects of pesticides on honey bees by considering the deformation rates in the control group. In future studies, the mechanisms of deformations in emerged honeybees that were identified here should be investigated.

## 5. Conclusions

Overall, there were no statistically significant differences in the emergence rates of adult bees between the horizontal and vertical plates, but the total deformation rates of the horizontal plates were significantly higher than those of the vertical plates. Our results are the first to discuss the emergence rates and deformation rates of honey bees concerning the position of plates in a laboratory. Considering these conclusions, the vertical rearing method with lower adult deformation rates appears to be more suitable, when considering the deformation rates of the control groups in order to verify the sublethal effects of pesticides on the bees. In the honey-bee larval toxicity test, according to the OECD guidelines, it is necessary to confirm the pupal mortality at the pupal stages (from D8 to D21). However, the vertical rearing plates must be capped with a wax layer on D15, so it may be difficult to check the pupal mortality after D15. Consequently, the rearing conditions and the position of rearing plates should be carefully considered depending on the purpose of the larval toxicity tests.

## Figures and Tables

**Table 1:** The volume and larval diet component percentages, according to the OECD guidelines [30,31].

Day	1	3	4, 5, 6
Volume of diet/larva (μL)	20	20	30, 40, 50
Diet component	Diet A	Diet B	Diet C
Royal jelly (%)	50.0	50.0	50.0
Distilled water (%)	37.0	33.5	30.0
Glucose (%)	6.0	7.5	9.0
Fructose (%)	6.0	7.5	9.0
Yeast extract (%)	1.0	1.5	2.0

**Table 2:** List and descriptions of mortality symptoms during developmental stage and deformation symptoms observed in emerged adult bees. This table is modified from Fine et al. [42] and Barbosa et al. [43].

Symptom	Description
Early death (ED)	Sunk in diet, failed to maintain the C-shape, flattening
Melanizing death (MD)	Death with darkening internally or externally, having black spots
Failed molt (FM)	Failure to evert imaginal discs, but the pupal molt is incomplete
Failed adult molt (FA)	Failure to emerge from final molt
Surviving normal (SN)	Survived and successfully eclosed as bees
Deformed wings (DW)	Eclosed as bees with deformed wings
-with short wings (DSW)	Eclosed as bees with short wings
-with tangled wings (DTW)	Eclosed as bees with tangled wings
Deformed antennae (DA)	Eclosed as bees with deformed antennae
Abnormal abdomen shape (AAS)	Eclosed as bees with abnormal abdomen shape (humpback)

**Table 3:** Larval mortality, pupal mortality, and adult emergence rate in *Apis mellifera*. Values are means ± SE. No significant differences in mortalities and emergence rates were found among the three groups (chi-square test, *p* > 0.05).

	NHG ^1^	CHG ^2^	CVG ^3^	*p* Values
Larval mortality (%)	4.9 (±0.7)	7.7 (±4.1)	4.2 (±2.7)	0.396
Pupal mortality (%)	16.1 (±2.8)	13.5 (±3.4)	15.3 (±2.2)	0.843
Adult emergence rate (%)	79.9 (±3.3)	79.9 (±6.7)	81.3 (±0.9)	0.943

^1^ NHG: the non-capped and horizontally oriented groups; ^2^ CHG: the capped and horizontally oriented groups; ^3^ CVG: the capped and vertically oriented groups.

**Table 4:** The overall percentage of observed symptoms of newly emerged adult bees. Each percentage in the following table is a calculated value of the ratio of the number of deformed bees to the total number of emerged bees.

Observed Symptoms ^1^	NHG ^2^	CHG ^3^	CVG ^4^
SN (%)	73.0	73.0	88.0
AAS (%)	13.9	4.3	3.4
DSW (%)	0.9	4.3	1.7
DTW (%)	0.9	3.5	1.7
DTWA (%)	3.5	6.1	3.4
DA (%)	0.0	0.9	0.9
AAS + DW (%)	4.3	4.3	0.0
AAS + DA (%)	0.0	1.7	0.0
DW + DA (%)	0.9	0.9	0.9
AAS + DW + DA (%)	2.6	0.9	0.0

^1^ SN: survived and successfully eclosed as bees; AAS: abnormal abdomen shape; DSW: deformed with short wings; DTW: deformed with tangled wings; DTWA: deformed with tangled wings asymmetrically; DA: deformed antennae; DW: deformed wings; ^2^ NHG: the non-capped and horizontally oriented groups; ^3^ CHG: the capped and horizontally oriented groups; ^4^ CVG: the capped and vertically oriented groups.

**Table 5:** The body weight and length of the newly emerged adult bees. Values are means ± SD. Means followed by the different letters across a row are significantly different (one-way ANOVA, *p* < 0.05).

	Weight (mg)	Length (mm)
NHG ^1^	67.40 (±12.24) a	10.88 (±0.96) a
CHG ^2^	69.77 (±12.63) ab	10.82 (±0.92) a
CVG ^3^	71.95 (±12.12) b	11.55 (±1.00) b

^1^ NHG: the non-capped and horizontally oriented groups; ^2^ CHG: the capped and horizontally oriented groups; ^3^ CVG: the capped and vertically oriented groups.
